# Identification of a stem-like cell population by exposing metastatic breast cancer cell lines to repetitive cycles of hypoxia and reoxygenation

**DOI:** 10.1186/bcr2773

**Published:** 2010-11-10

**Authors:** Elizabeth Louie, Sara Nik, Juei-suei Chen, Marlies Schmidt, Bo Song, Christine Pacson, Xiu Fang Chen, Seonhye Park, Jingfang Ju, Emily I Chen

**Affiliations:** 1 Department of Pharmacological Sciences, Stony Brook University, BST-125, Stony Brook, NY 11794, USA; 2 Department of Pathology, Stony Brook University Medical Center, Stony Brook, NY 11794, USA

## Abstract

**Introduction:**

The irregular vasculature of solid tumors creates hypoxic regions, which are characterized by cyclic periods of hypoxia and reoxygenation. Accumulated evidence suggests that chronic and repetitive exposure to hypoxia and reoxygenation seem to provide an advantage to tumor growth. Although the development of hypoxia tolerance in tumors predicts poor prognosis, mechanisms contributing to hypoxia tolerance remain to be elucidated. Recent studies have described a subpopulation of cancer stem cells (CSC) within tumors, which have stem-like properties such as self-renewal and the ability to differentiate into multiple cell types. The cancer stem cell theory suggests CSCs persist in tumors as a distinct population and cause relapse and metastasis by giving rise to new tumors. Since hypoxia is considered to be one of the critical niche factors to promote invasive growth of tumors, we hypothesize that repetitive cycles of hypoxia/reoxygenation also play a role in the enrichment of breast CSCs.

**Methods:**

Two metastatic human breast cancer cell lines (MDA-MB 231 and BCM2) were used to optimize the conditions of hypoxia and reoxygenation cycles. The percentage of CSCs in the cycling hypoxia selected subpopulation was analyzed based on the CD44, CD24, ESA, and E-cadherin expression by three-color flow cytometry. Colony formation assays were used to assess the ability of this subpopulation to self-renew. Limiting dilution assays were performed to evaluate the tumor-initiating and metastatic ability of this subpopulation. Induction of EMT was examined by the expression of EMT-associated markers and EMT-associated microRNAs.

**Results:**

Using an optimized hypoxia and reoxygenation regimen, we identified a novel cycling hypoxia-selected subpopulation from human breast cancer cell lines and demonstrated that a stem-like breast cancer cell subpopulation could be expanded through repetitive hypoxia/reoxygenation cycles without genetic manipulation. We also found that cells derived from this novel subpopulation form colonies readily, are highly tumorigenic in immune-deficient mice, and exhibit both stem-like and EMT phenotypes.

**Conclusions:**

These results provide the validity to the newly developed hypoxia/reoxygenation culture system for examining the regulation of CSCs in breast cancer cell lines by niche factors in the tumor microenvironment and developing differential targeting strategies to eradicate breast CSCs.

## Introduction

Recent studies have described a subpopulation of cancer cells within tumors termed 'cancer stem cells' (CSCs), which have stem-like properties such as self-renewal and the ability to differentiate into multiple cancer cell types [1-7]. The CSC theory suggests that such CSCs persist in tumors as a distinct population and cause relapse and metastasis by giving rise to new tumors [8-10]. Although CSCs make up only a small fraction of a tumor, they possess the unique capability to regenerate a tumor whereas most tumor cells lack this regenerative capability [11,12]. By means of a non-obese diabetic/severe combined immunodeficiency disease (NOD/SCID) xenotransplant assay in combination with specific cell surface markers (CD44^+^CD24^-/low^), CSCs were enriched from metastatic and primary breast tumors and were shown to have the ability to reestablish tumor heterogeneity after transplantation [1]. Since then, additional CSC markers have been proposed and studied to isolate putative tumor stem cell populations. However, as demonstrated by a recent report from Stuelten and colleagues [13], the complexity of CSC markers continues to pose challenges for identifying and isolating the putative tumor stem cell populations by the cell-sorting approach. In addition to initiating tumors, CSCs are thought to be capable of initiating metastasis. The link between CSCs and metastasis has been suggested by several studies. First, breast CSCs were shown to invade through Matrigel, a basement membrane matrix used routinely as an indicator of metastatic potential of cancer cells [14]. Second, a recent study demonstrated that there is a link between epithelial-mesenchymal transition (EMT) and breast CSCs [15]. Furthermore, the prevalence of CD44^+^CD24^- ^cells in breast cancer patients indicates a link between high numbers of stem-like cancer cells and metastasis [16]. However, only a few studies have directly tested the metastatic capability of putative CSCs *in vivo*. Collective evidence from a few studies that directly tested the *in vivo *metastasis using sorted CSCs suggests that the CSC phenotype alone may exhibit invasive property *in vitro *but is inadequate to determine or predict *in vivo *metastasis. For example, in pancreatic cancer, CSCs (CD133^+ ^cells) were not able to metastasize when injected orthotopically at low numbers [17]. In mammary carcinomas, CD44^+^CD24^low ^cells were invasive *in vitro *but the phenotype was not sufficient for metastasis when cells were injected intracardiacally *in vivo *[14]. Therefore, we set out to investigate alternative mechanisms that could enrich for breast CSCs with tumor-initiating and metastatic capabilities.

To identify factors that distinguish the malignant subpopulation within breast tumors, we began to explore environmental influences known to associate with aggressively metastatic breast tumors. It has been postulated that hypoxia contributes directly to the development of more aggressive cancers by exerting selective pressure on the tumor cell population to favor cells that can survive decreased O_2 _and nutrients [18-20]. During tumor development, rapid expansion of cancer cells creates a hypoxic microenvironment that is followed by periods of reoxygenation to promote tumor progression. These two aspects of tumor progression (hypoxia and reoxygenation) cooperate to provide growth advantages essential for the progressive development of aggressive tumors [21]. Although extensive efforts have been devoted to understanding the effect of hypoxia on tumor progression, two areas of tumor biology remain unclear. What is the effect of fluctuating oxygen tension on tumor progression? How does hypoxia drive an irreversible phenotype without genetic manipulation? It is known that both hypoxia and consecutive hypoxia/reoxygenation can exert a variety of effects on tumor cell biology, including activation of pro-survival signal transduction pathways, aberrant genetic and epigenetic alterations, and increased tumor angiogenesis. In some studies, hypoxia/reoxygenation was shown to drive expression of proteins associated with poor prognosis [22]. Furthermore, susceptibility of genomic instability can vary after acute or chronic exposure to hypoxia followed by reoxygenation [23]. Therefore, we hypothesize that hypoxia/reoxygenation cycles may provide the driving force to select for a highly metastatic breast CSC subpopulation.

To study the effect of hypoxia/reoxygenation cycles on breast cancer, we exposed two metastatic human breast cancer cell lines (MDA-MB 231 and BCM2) to cycles of chronic hypoxia and nutrient deprivation. After one cycle of hypoxia and reoxygenation, we observed a small cell population that survived hypoxia as spherical clusters under hypoxic conditions and that resumed proliferation after reoxygenation. We then isolated and exposed this novel subpopulation to additional cycles of hypoxia/reoxygenation and established a distinct subpopulation of cells from these two breast cancer cell lines. As expected, this novel subpopulation showed increasing viability under hypoxia and the ability to proliferate as either an adherent monolayer or substrate-independent tumor spheres. Interestingly, an increased fraction of the cell population was found to express CD44^+^/CD24^-^/ESA^+ ^cell surface markers. However, this novel subpopulation was distinguished by being highly tumorigenic and metastatic and showed upregulation of EMT markers. These findings strongly suggest that we have succeeded in isolating a unique metastatic CSC population by exposing breast cancer cells to repetitive cycles of hypoxia and reoxygenation.

## Materials and methods

### Cell lines and tissue culture

MDA-MB 231 was purchased from American Type Culture Collection (Manassas, VA, USA); BCM2 cell line was obtained from Brunhilde Felding-Habermann (Scripps Research Institute, La Jolla, CA, USA). MDA-MB 231 and BCM2 cell lines were cultured in minimum essential medium (MEM) (Invitrogen Corporation, Carlsbad, CA, USA) supplemented with 10% fetal bovine serum (HyClone, Logan, UT, USA; Thermo Fisher Scientific Inc., Waltham, MA, USA), 2 mM L-glutamine, 1 mM sodium pyruvate, 1 mM non-essential amino acids, and 1% vitamin (HyClone). All cell lines were grown at 37°C and in 5% carbon dioxide.

### Flow cytometry

Non-confluent cultures were trypsinized into single-cell suspension, counted, washed with phosphate-buffered saline (PBS), and fixed with 10% paraformaldehyde. Cells were stained with antibodies specific for human cell surface markers: CD326/ESA-FITC, CD24-PE, and CD44-PE-Cy7 (BD Pharmingen, San Jose, CA, USA). E-cadherin antibody was purchased from Cell Signaling Technology, Inc. (Danvers, MA, USA). A total of 2 × 10^5 ^cells were incubated with antibodies for 30 minutes on ice. Unbound antibody was washed, and cells were analyzed on a Guava EasyCyte Plus Flow Cytometer (Millipore Corporation, Billerica, MA, USA). The Student *t *test was used to calculate the significance of increased CD44^+^/CD24^-^/ESA^+ ^and E-cad^-^/CD44^+^/CD24^- ^populations in the cycling hypoxia-selected subpopulations.

### Viability and cell proliferation assay

Cells were collected from either the media (floating subpopulation) or the adherent subpopulation after each hypoxic cycle and dissociated into single-cell suspension by trypsin/EDTA (trypsin/ethylenediaminetetraacetic acid). Guava ViaCount Reagent (Millipore Corporation) was used to determine the viability and cell numbers after each hypoxic cycle. For cell proliferation assay, 5 × 10^4 ^cells were seeded in triplicate wells per cell line per time point in 12-well tissue culture plates. Cells were detached (adherent culture) or dissociated (spherical culture) by trypsin/EDTA and resuspended in PBS for cell counting using the ViaCount assay. In accordance with the manufacturer's instructions, an appropriate volume of the ViaCount Reagent was mixed with 50 μL of cell suspension in PBS. After 5 minutes of incubation at room temperature in the dark, ViaCount/cell mixture was loaded into a 96-well microtiter plate and read by the Guava Flow Cytometer. Both live and dead cells were detected and displayed in dot plots. The Guava ViaCount software carried out calculation of viability and cell numbers for each sample automatically.

### Colony-forming assays

For the colony formation assay, cells were trypsinized to generate single-cell suspensions and counted by a hemocytometer. Single-cell suspensions (500 cells per well) were plated on 96-well plates with an ultralow attachment surface. Three wells were seeded for each cell line, and triplicate experiments were performed per cell line (*n *= 3). The EVOS microscope (Advanced Microscopy Group, Bothell, WA, USA) was used to count tumor spheres and take images of each well on days 1, 3, 5, and 9. Immediately after the cells were seeded, each well was checked under the microscope to verify the sparseness of each spherical culture, and only wells containing single-cell suspension with no cell cluster were chosen for tumor-sphere counting after 9 days. Colonies of at least 60 μm in diameter (determined by using an eyepiece graticule with crossed scales) were counted on day 9 after plating. Colony-forming efficiency (CFE) was calculated by dividing the number of colonies (> 60 μm) formed by the original number of single cells seeded and is expressed as a percentage.

### Animals and surgery

All animal procedures were performed in accordance with an approved protocol by the Stony Brook University Institutional Animal Care and Use Committee. NOD/SCID mice were purchased from the Jackson Laboratory (Bar Harbor, ME, USA). Four- to six-week-old female mice were used for tumor injections. Human breast cancer cells were suspended in sterile Hank's buffered salt solution (HBSS) and injected into the third thoracic mammary gland. Tumor formation was assessed by palpation at least once a week. A caliper was used to measure the length and width of tumors at least once a week. A standard formula for calculating tumor volume (in cubic millimeters) was used: length × (width)^2^/2. Duplicate experiments were performed for each cell number.

### Quantification of lung metastasis

Five to six weeks after the resection of primary tumors from tumor-bearing NOD/SCID mice, lungs were harvested and fixed in the Bouin's fixative solution overnight. Nodules on the surface of lungs were visualized under the dissecting microscope, and a USB digital camera (Leica Microsystems, Bannockburn, IL, USA) from the microscope was used to take gross images of each lung. To quantify the tumor burden, each image was first annotated with the National Institutes of Health ImageJ software to identify measurable nodules on the surface of the lung. The length and width of each nodule were measured with pixels and then converted to millimeters to calculate the tumor volume. A standard formula for calculating tumor volume (cubic millimeters) was used: length × (width)^2^/2. Metastatic tumor burden of each animal was calculated by combining tumor volumes from four lobes of lungs. Average tumor burden and standard deviations were derived from at least four animals per group.

### Western blot analysis

Subcellular fractionation of cell lysates was prepared using the ProteoExtract Subcellular Proteome Extraction Kit (Calbiochem-EMD Biosciences, San Diego, CA, USA), and protein concentrations were determined by EZQ Protein Quantification Kit (Invitrogen Corporation). Proteins from nuclear or cytoplasmic fractions (40 μg) were resolved by 4% to 20% SDS-PAGE and transferred onto nitrocellulose membranes for immunoblotting. Immunoblotting assays were carried out by standard procedures using Snail (Cell Signaling Technology, Inc.) and vimentin antibodies (Novus Biologicals, Littleton, CO, USA). Anti-β-actin antibody (Sigma-Aldrich, St. Louis, MO, USA) was used to confirm equal protein loading of cytoplasmic fractions. Anti-histone H3 antibody (Millipore Corporation) was used to confirm equal protein loading of nuclear fractions. Secondary antibodies conjugated with either AF680 (Invitrogen Corporation) or CW800 (LI-COR Biosciences, Lincoln, NE, USA) were used to visualize protein bands using the Odyssey Infrared Imager (LI-COR Biosciences).

### Analysis of the mRNA and microRNA expression by quantitative reverse transcription-polymerase chain reaction assay

Total RNA was isolated from each cell line using an RNA Extraction Kit (Qiagen Inc., Valencia, CA, USA) in accordance with the manufacturer's instructions. One point two micrograms of total RNA from each cell line was reverse-transcribed using random primers and the High-Capacity cDNA Synthesis Kit (Applied Biosystems, Foster City, CA, USA). The resulting cDNAs were mixed with the SYBR PCR [polymerase chain reaction] master mix (Applied Biosystems) and run on the StepOnePlus Applied Biosystems Real-time PCR machine. One cycle of denaturing step (10 minutes at 95°C) was applied, followed by 35 cycles of amplification (15 seconds at 95°C and 1 minute at 58°C), with fluorescence measured during the extension. Primers used to amplify the human *Snail *gene have the following sequences: 5'-CCTCCCTGTCAGATGAGGAC-3' (forward) and 5'-CCAGGCTGAGGTATTCCTG-3' (reverse). Primers used to amplify the human *Slug *gene have the following sequences: 5'-GGGGAGAAGCCTTTTTCTTG-3' (forward) and 5'-TCCTCATGTTTGTGCAGGAG-3' (reverse). Primers used to amplify the human *Twist *gene have the following sequences: 5'-GGAGTCCGCAGTCTTACGAG-3' (forward) and 5'-TCTGGAGGACCTGGTAGAGG-3' (reverse). The relative quantification (RQ) value reflects the fold changes of mRNA expression in each cell line compared with the parental cell lines. RQ was calculated using the comparative *C*_T _(ΔΔ*C*_T_) method and StepOne software version 2.0.1 (Applied Biosystems) and normalized by the expression of the housekeeping gene, *GAPDH *(glyceraldehyde-3-phosphate dehydrogenase). Three independent experiments were performed to derive average RQ and standard deviations (*n *= 3).

For microRNA (miRNA) analysis, total RNAs, including miRNAs, were isolated from the cell lines using TRIzol reagent (Invitrogen Corporation) in accordance with the manufacturer's instructions. Ten nanograms of total RNA from each cell line was converted to cDNA using the High-Capacity cDNA synthesis kit (Applied Biosystems). The miRNA sequence-specific reverse transcription-PCR (RT-PCR) primers for miR-200c, miR-205, and an endogenous control RNU6B were purchased from Ambion (Austin, TX, USA). Real-time quantitative RT-PCR (qRT-PCR) analysis was performed using the 7500 Real-Time PCR System from Applied Biosystems. The TaqMan master mix was added to the cDNA and primer mix to amplify and quantify target miRNAs (No AmpErase UNG; Applied Biosystems). The following PCR cycle was used for miRNA amplifications: 95°C for 10 minutes and 40 cycles of 95°C for 15 seconds and 60°C for 60 seconds. The RQ value reflects the fold changes of miRNA expression in each cell line compared with the parental cell lines. RQ was calculated using the comparative *C*_T _(ΔΔ*C*_T_) method and StepOne software version 2.0.1 (Applied Biosystems) and normalized by the expression of the internal control RNU6B. Three independent experiments were performed to derive average RQ and standard deviations (*n *= 3).

## Results

### Establishing optimized conditions for hypoxia and reoxygenation selection

**Figure 1:**
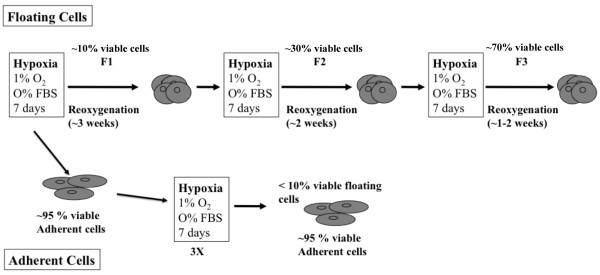
**An illustration of the hypoxia and reoxygenation regimen**. Conditions for hypoxia/reoxygenation cycles were optimized using two human metastatic breast cancer cell lines (MDA-MB 231 and BCM2). The viability of non-adherent cells is listed after each hypoxic cycle. FBS, fetal bovine serum.

To study the effect of repetitive hypoxia/reoxygenation on breast cancer cells, we optimized conditions of hypoxia and reoxygenation with regard to the number of cycles, exposure time, cell numbers, and other parameters. We used two metastatic breast cancer cell lines, MDA-MB 231 and BCM2 [24], to ensure that our current protocol was not cell line-specific. To establish efficient and reproducible hypoxia/reoxygenation conditions, we used the ProOxC system (BioSpherix, Lacona, NY, USA), which allows precise oxygen control over an extended time period via an oxygen sensor and automatic feedback mechanisms to maintain a consistently low oxygen environment. The optimized procedure included exposing these breast cancer cell lines (1 × 10^7 ^cells in a T125 tissue culture flask, approximately 60% confluency) to hypoxia (1% O_2_) and nutrient deprivation for 7 days, mimicking the avascular microenvironment, and reoxygenating surviving cells for 1 to 3 weeks depending on the viability of surviving cells (Figure 1). The same growth media used to maintain monolayer cultures (see Materials and methods) were used to prepare the cells and maintain the adherent and non-adherent cells in culture. After each hypoxic cycle, 10% of the adherent culture became non-adherent (floating cell population). Using this protocol, we consistently observed novel surviving populations that were non-adherent and that demonstrated a propensity to form spherical clusters under hypoxic conditions. However, unlike the adherent hypoxia-resistant cells, only 10% of the total non-adherent cells were viable after the first round of hypoxic cycle (F1). After reoxygenating and growing F1 cells as spherical cultures, we exposed the same amount of F1 cells to a second hypoxic cycle and found an increase in viability (30%) (F2). After three rounds of hypoxia and oxygenation, we found that the viability of the non-adherent cells increased to 70% (F3). Additional rounds of hypoxia did not increase the percentage of survival significantly. In addition to finding increased hypoxia viability, we found distinct characteristics in this novel subpopulation. For comparison purposes, we exposed the adherent hypoxia-resistant cells to two additional rounds of hypoxia/reoxygenation selection and generated the hypoxia-exposed adherent cell population (A3).

### The newly isolated cycling hypoxia-selected breast cancer subpopulation has increased tumor-initiating capability

**Figure 2:**
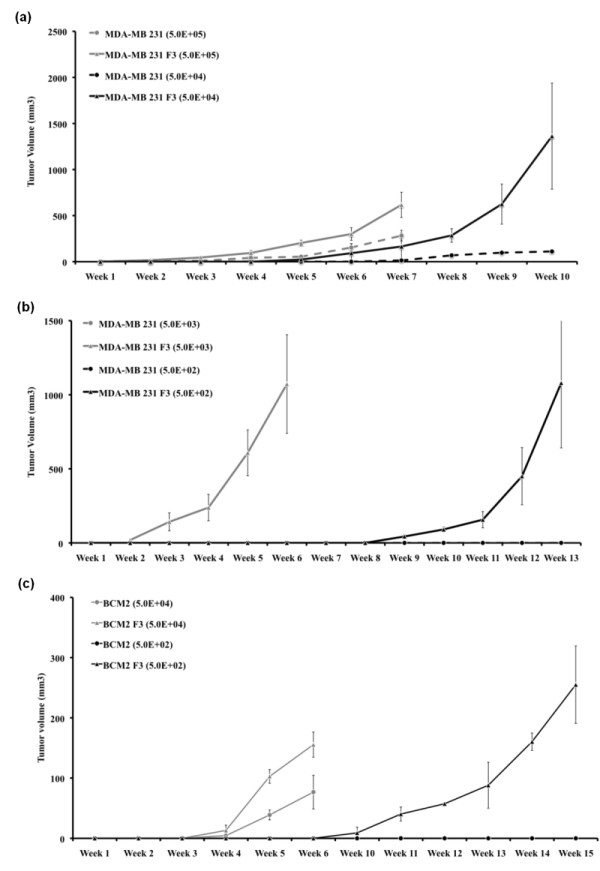
**Growth curves of primary tumors generated from the parental cell lines and cycling hypoxia-selected subpopulations**. **(a) **Growth curves of primary tumors in non-obese diabetic/severe combined immunodeficiency disease (NOD/SCID) mice injected with 5 × 10^5 ^or 5 × 10^4 ^MDA-MB 231 and MDA-MB 231 F3 cells. Tumors were first detected by palpation. Standard errors are derived from duplicate experiments (*n *= 6 per cell number for each experiment). **(b) **Growth curves of tumors in NOD/SCID mice injected with 5 × 10^3 ^or 5 × 10^2 ^MDA-MB 231 and MDA-MB 231 F3 cells. Standard errors were derived from duplicate experiments (*n *= 5 per cell number for each experiment). **(c) **Growth curves of tumors in NOD/SCID mice injected with 5 × 10^4 ^or 5 × 10^2 ^BCM2 and BCM2 F3 cells. Standard errors were derived from duplicate experiments (*n *= 5 per cell number for each experiment).

Since hypoxia has been implicated in the promoting of aggressive tumors, we first examined the tumor-initiating ability of the parental and newly isolated cycling hypoxia-selected subpopulation in immune-deficient mice. We orthotopically injected either parent MDA-MB 231 and BCM2 or the cycling hypoxia-selected subpopulations (MDA-MB 231 F3 and BCM2 F3) into NOD/SCID mice as a limiting dilution assay from 5 × 10^5 ^to 5 × 10^2 ^cells per mammary gland. Although there was no noticeable difference in the frequency of tumor formation between these two cell populations (MDA-MB 231 and MDA-MB 231 F3) when 5 × 10^5 ^cells were injected, a dramatic difference in tumor initiation and growth was observed when 10-fold, 100-fold, and 1,000-fold fewer cells were injected in NOD/SCID mice (Figure 2a). With as few as 500 cells, MDA-MB 231 F3 and BCM2 F3 cells could form tumors in NOD/SCID mice, whereas both parent cell lines were not able to do so at the same dilution (Table 1 and Figure 2b,c). Furthermore, tumors progressed rapidly in mice injected with lower numbers of MDA-MB 231 F3 cells and BCM2 F3 cells (5 × 10^4 ^cells) than equivalent numbers of MDA-MB 231 cells (Figure 2a-c), indicating that MDA-MB 231 F3 cells formed more aggressive tumors. We also injected 500 hypoxia-exposed adherent cells (MDA-MB 231 A3 and BCM2 A3) in the mammary fat pad of female NOD/SCID mice. None of the mice injected with 500 MDA-MB 231 A3 or BCM2 A3 cells developed mammary tumors by 90 days (MDA-MB 231 A3) or 98 days (BCM2 A3) (Table 1). We conclude that the newly isolated cycling hypoxia-selected non-adherent subpopulation is highly tumorigenic compared with the hypoxia-exposed adherent and parental cell lines.

**Table 1:** Limiting dilution tumor formation of parental, hypoxia-exposed adherent, and cycling hypoxia-selected breast cancer cells *in vivo*

Cell type	Days	Number injected and tumors formed			
		
		**5 × 10**^**5**^	**5 × 10**^**4**^	**5 × 10**^**3**^	**5 × 10**^**2**^
MDA-MB 231 (total cell population)	24-90	5/6	2/6	0/5	0/5
MDA-MB 231 A3 (hypoxia-exposed adherent cells)	90	-	-	-	0/5
MDA-MB 231 F3 (cycling hypoxia-selected subpopulation)	10-90	6/6	6/6	5/5	4/5
BCM2 (total cell population)	35-98	-	5/5	-	0/5
BCM2 A3 (hypoxia-exposed adherent cells)	98	-	-	-	0/5
BCM2 F3 (cycling hypoxia-selected subpopulation)	28-98	-	5/5	-	4/5

### The newly isolated cycling hypoxia-selected breast cancer subpopulation comprises putative breast cancer stem cell population (CD44^+^/CD24^-^/ESA^+^)

**Figure 3:**
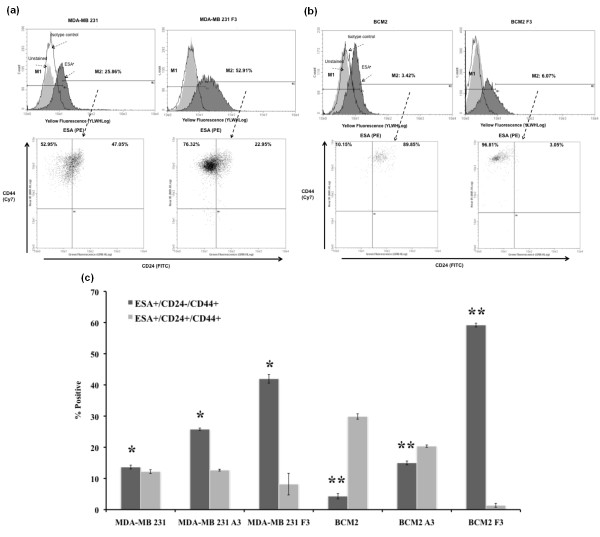
**Cell surface expression of ESA, CD44, and CD24**. Three-color flow cytometry analysis was performed to detect the CD44^+^/CD24^-^/ESA^+ ^cell population. **(a) **Top panel: Unstained, isotype control, and ESA-stained cells are shown in the histogram. ESA staining of MDA-MB 231 (total cell population) and MDA-MB 231 F3 (cycling hypoxia-selected subpopulation) cells. M1 marker gates ESA^+ ^cells, and M2 marker gates the ESA^- ^cells. Bottom panel: CD44 and CD24 expression of ESA^+ ^cells in each cell line. The percentages of the CD44^+^/CD24^- ^and CD44^+^/CD24^+ ^cells within the ESA^+ ^cell population are indicated in the Quad plot. **(b) **The same flow cytometry analysis for BCM2 (total cell population) and BCM2 F3 (cycling hypoxia-selected subpopulation) cells. **(c) **Quantitative comparison of CD44^+^/CD24^-^/ESA^+ ^cell population and CD44^+^/CD24^+^/ESA^+ ^cell population in cycling hypoxia-selected subpopulations (MDA-MB 231 F3 and BCM2 F3) and their parental breast cancer cell lines. At least three replications were performed to derive the average percentage of each cell population in each cell line and standard deviations. Asterisks indicate statistical significance by two-tail *t *test (*n *= 3, *P *< 0.05). ESA, epithelial-specific antigen.

Evidence of stem-like cancer cells has been established in various types of cancer, including breast cancer [1,3,4,7,8,12,25]. This small subpopulation has been shown to be highly tumorigenic and exhibits some stem cell characteristics such as self-renewal and the ability to form tumor spheres [12,26]. Since the newly isolated cycling hypoxia-selected subpopulation is highly tumorigenic, we hypothesized that this subpopulation is enriched with stem-like breast cancer cells. Using the three-color flow cytometry analysis, we found that the cycling hypoxia-selected subpopulation displayed an increased proportion of cells with the surface marker expression of CD44^+^/CD24^-^/ESA^+^, which has been reported as putative breast CSCs. The percentage of the CD44^+^/CD24^-^/ESA^+ ^cell population was significantly increased in the cycling hypoxia-selected subpopulation in comparison with the parental breast cancer cell lines (3-fold increase in MDA-MB 231 F3 cells, Figure 3a,c; 13-fold increase in BCM2 F3, Figure 3b,c; Figure S1 in Additional file 1). Since it has been reported that hypoxia exposure alone can increase the stem-like cell population, we also examined the percentage of CD44^+^/CD24^-^/ESA^+ ^and CD44^+^/CD24^+^/ESA^+ ^cell populations in the hypoxia-exposed adherent cells (MDA-MB 231 A3 and BCM2 A3) and found only a slight increase in the percentage of CD44^+^/CD24^-^/ESA^+ ^cell population in the hypoxia-exposed adherent cells compared with the parental breast cancer cell lines (1.8-fold increase in MDA-MB 231 A3 cells, Figure 3c; 3.5-fold increase in BCM2 A3, Figure 3c). Thus, our data suggest that cyclic exposures of hypoxia and reoxygenation are crucial for the enrichment of stem-like breast cancer cells in this novel non-adherent subpopulation.

**Figure 4:**
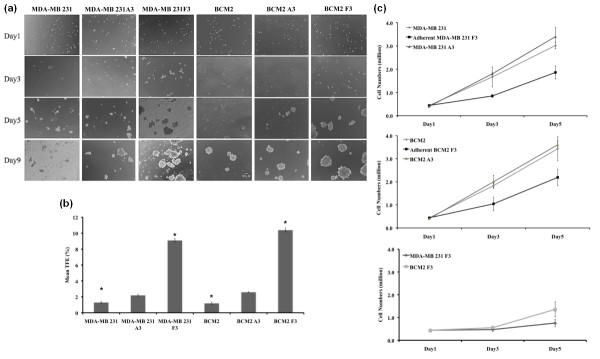
**The colony formation and proliferation of the parent cell lines and the cycling hypoxia-selected subpopulations**. **(a) **Images of colony formation assays from 500 cells of MDA-MB 231, MDA-MB 231 A3, MDA-MB F3, BCM2, BCM2 A3, and BCM2 F3 on days 1, 3, 5, and 9. Representative images from three separate experiments are shown. **(b) **Colony-forming efficiency (CFE) of parental cells, hypoxia-exposed adherent cells (MDA-MB 231 A3 and BCM2 A3), and the cycling hypoxia-selected subpopulations (MDA-MB 231 F3 and BCM2 F3). CFE was determined when tumor spheres were larger than 60 μm in diameter on day 9 after plating. CFE was calculated by dividing the number of tumor spheres formed by the original number of single cells seeded and is expressed as a percentage. Three independent experiments were performed to derive the average CFE and standard deviation per cell line. Asterisk indicates statistical significance by two-tail *t *test (*n *= 3, *P *< 0.05). **(c) **Proliferation curves of the parental cells, hypoxia-exposed cells (A3), and the cycling hypoxia-selected subpopulations grown as adherent cultures (top two plots) or as spherical cultures (the third plot). Total viable cells from each well at days 3 and 5 were counted by the ViaCount assay using the Guava Flow Cytometer. Average cell numbers and standard deviations at each time point were calculated from three independent experiments.

It has been shown that tumor-initiating cells or CSCs resemble stem cells in their ability to grow as spheres when cultured under conditions in which they cannot attach to a solid substratum [6,27]. To test whether the increased tumor-initiating capability observed in the cycling hypoxia-selected subpopulations correlates with their ability to grow as tumor spheres in culture, cells from parental cell populations (MDA-MB 231 and BCM2), hypoxia-exposed adherent cells (MDA-MB 231 A3 and BCM2 A3), and cycling hypoxia-selected subpopulations (MDA-MB 231 F3 and BCM2 F3) were plated at low density (500 cells per 0.32 cm^2^) onto ultralow attachment plates in MEM. Images of cells from each well were taken at days 1, 3, 5, and 9 to visualize the formation of colonies. After day 5, we noticed that the appearance of compact and asymmetric solid tumor spheres developed in all wells containing MDA-MB 231 F3 and BCM2 F3 cells whereas only small clusters of cells were found in wells containing MDA-MB 231 and BCM2 cells (Figure 4a). By day 9, we observed that some large tumor spheres began to form in the parental cell lines. The time line of tumor-sphere formation was comparable between hypoxia-exposed adherent cells (MDA-MB 231 A3 and BCM2 A3) and their parental cell lines. To determine the CFE of each cell population, tumor spheres of at least 60 μm were counted on day 9 after plating. CFE was calculated by dividing the number of tumor spheres formed by the original number of single cells seeded and was expressed as a percentage. Three independent experiments were performed to derive the average CFE and standard deviation per cell line. Although there is around a 2-fold increase in CFE in hypoxia-exposed cells (MDA-MB 231 A3 and BCM2 A3) compared with the parental cell lines, a larger increase (7- to 8-fold) of CFE was found in the cycling hypoxia-selected subpopulations compared with the parental cell lines (Figure 4b). Therefore, according to our results, the increased tumor-initiating capability of the cycling hypoxia-selected subpopulations is in accordance with the increased ability to grow as spherical colonies in culture.

In addition to showing increased ability to grow as spherical colonies, the stem-like cancer cells were shown to proliferate slower than the bulk cancer cells [28,29]. This attribute of stem-like cancer cells has significant clinical implications such as chemoresistance and radioresistance. To determine whether the cycling hypoxia-selected subpopulations cycle slower than the parental and hypoxia-exposed adherent cells, equal numbers of cells from each cell population were plated on day 1, and an increase in cell numbers over 5 days was used to assess the rate of proliferation. To compare the rate of proliferation under the same condition as the parental and hypoxia-exposed adherent cells, we plated the cycling hypoxia-selected subpopulation as adherent cells (adherent MDA-MB 231 F3 and adherent BCM2 F3) and measured the increase in cell numbers together with the other adherent cell populations. The proliferation of the cycling hypoxia-selected subpopulation as spherical culture was performed separately. While the parental and hypoxia-exposed adherent cells display similar rates of proliferation, the cycling hypoxia-selected subpopulations grow slower as adherent cells and even slower as suspension cultures (Figure 4c). Together, results from established assays for CSC activity show that the cycling hypoxia-selected subpopulation has an increased ability of tumor initiation and tumor-sphere formation. More importantly, these findings suggested that our hypoxia/reoxygenation scheme resulted in an enrichment of bona fide breast CSCs without selecting for user-defined markers or targeted genetic manipulation.

### The newly isolated cycling hypoxia-selected breast cancer subpopulation is highly metastatic and exhibits increasing epithelial-mesenchymal transition phenotype

Aside from their role in tumor initiation, stem-like cancer cells have been hypothesized to contribute directly to cancer metastasis. However, the collective evidence from a few studies examining the metastatic potential of CSCs *in vivo *suggests that CSC phenotype alone is not enough to determine metastasis. Particularly, a study by Sheridan and colleagues [14] demonstrates that CD44^+^/CD24^- ^stem-like phenotype is not sufficient for homing and proliferation at sites of metastasis, despite a display of increased invasive property *in vitro*.

**Figure 5:**
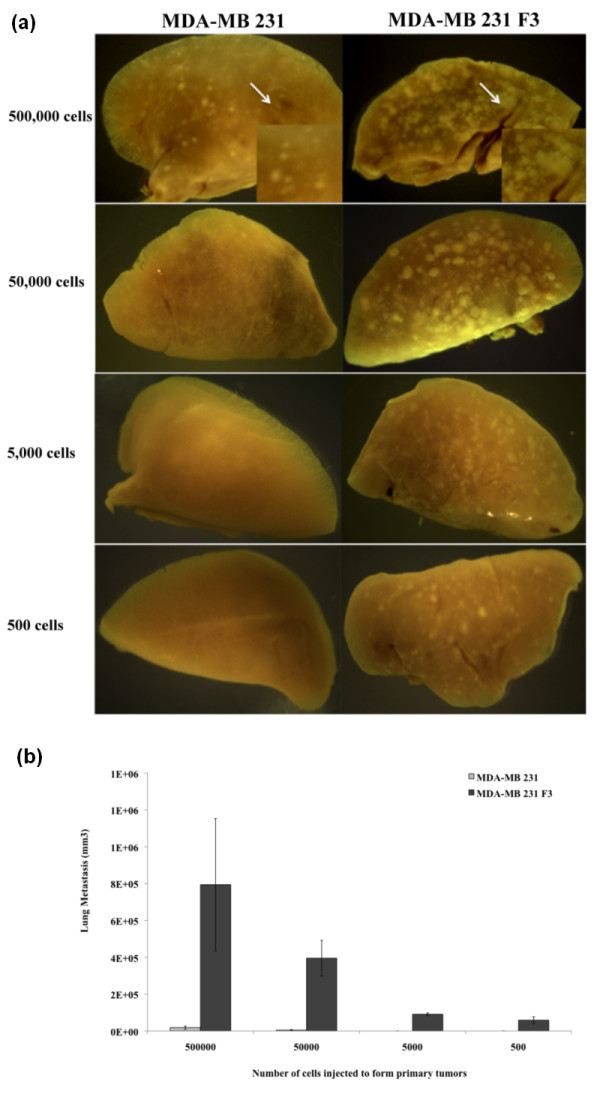
**Lung metastases from the tumor-initiating assays**. **(a) **Representative images of lungs from tumor-bearing non-obese diabetic/severe combined immunodeficiency disease (NOD/SCID) mice are shown (*n *= 6). A higher magnification of lung metastases derived from orthotopic injection of 5 × 10^5 ^MDA-MB 231 or MDA-MB 231 F3 cells is shown in the lower right corner. **(b) **Quantitative analysis of the metastatic tumor burden in the lung. Images of lungs were taken using a digital microscope camera. Images were imported into the National Institutes of Health ImageJ software for quantitative analysis. The sum of metastatic tumor volume was first calculated from four lobes of lungs per animal (see Materials and methods), and the lung metastasis per group is presented as tumor volume (cubic millimeters). The number of animals used per group is listed in Table 1.

Using the human cancer cell xenograft model, we were able to quantify lung metastases 4 weeks after the surgical resection of primary tumors. Images of lungs were taken by a digital camera built into the microscope and were used for quantifying metastases. The National Institutes of Health ImageJ software was used to measure the diameters (width and length) of each visible metastasis on the lung, and total tumor volumes were calculated from each animal (for a description, see Materials and methods). Overall, lungs harvested from mice with primary tumors derived from the cycling hypoxia-selected MDA-MB 231 F3 cells showed significantly more metastases than lungs harvested from mice with parental cell primary tumors (Figure 5a). Mice injected with 5 × 10^5 ^MDA-MB 231 F3 cells showed a 43-fold greater lung tumor burden, by volume, than mice injected with 5 × 10^5 ^parental cells (Figure 5b). Mice injected with 5 × 10^4 ^MDA-MB 231 F3 cells had 76-fold greater lung tumor burden than mice injected with 5 × 10^4 ^parental cells (Figure 5b). Remarkably, mice injected with 100-fold and 1,000-fold less MDA-MB 231 F3 cells showed a less than 10-fold reduction in metastatic tumor burden, whereas this number of parental cells failed to form substantial metastases, indicating that the cycling hypoxia-selected subpopulation is highly metastatic *in vivo*.

**Figure 6:**
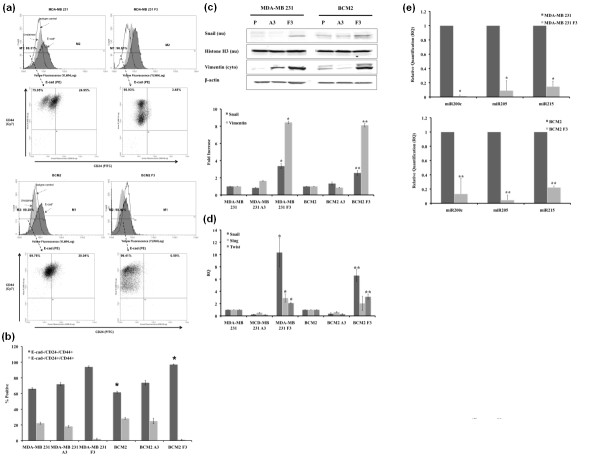
**Expression of epithelial-mesenchymal transition (EMT)-associated markers in the total cell population and the cycling hypoxia-selected subpopulation**. **(a) **Three-color fluorescence-activated cell sorting (FACS) analysis was performed to detect CD44CD24 expression profile on the E-cadherin-negative cell population. Unstained, isotype control, and E-cadherin (E-cad)-stained cells are shown in the histogram. ESA staining of MDA-MB 231 (total cell population) and MDA-MB 231 F3 (cycling hypoxia-selected subpopulation) cells is shown. M1 marker gates E-cadherin-positive cells, and M2 marker gates the E-cadherin-negative cells. Bottom panel: CD44 and CD24 expression of E-cadherin-negative cells in each cell line. The percentages of the CD44^+^/CD24^- ^and CD44^+^/CD24^+ ^cells are indicated in the Quad plot. **(b) **Quantitative comparison of E-cad^-^/CD44^+^/CD24^- ^cell population and E-cad^-^/CD44^+^/CD24^+ ^cell population in cycling hypoxia-selected subpopulations (MDA-MB 231 F3 and BCM2 F3) and their parental breast cancer cell lines. At least three replications were performed to derive the average percentage of each cell population in each cell line and standard deviations among the replicates. **(c) **Expression of EMT-associated proteins in the parental cells (P), hypoxia-exposed adherent cells (A3), and the cycling hypoxia-selected subpopulations (F3). Lysates from these two cell populations were fractionated on the basis of the subcellular localization using the ProteoExtract Kit, and 40 μg of proteins was loaded for Western blotting. Beta-actin is used as the loading control for the cytoplasmic fraction, and histone H3 is used as the loading control for the nuclear fraction. Western blot images (*n *= 3) are quantified by National Institutes of Health ImageJ software and presented as fold increase in A3 and F3 cells compared with the parental cell lines. **(d) **mRNA regulation of EMT transcription factors in the parental, A3, and F3 cells. Quantitative reverse transcription-polymerase chain reaction (qRT-PCR) analyses were performed using specific primers to measure the mRNA expression of human Snail, Slug, and Twist genes in these three cell populations. At least three replications (*n *= 3) were performed to derive the average percentage of each cell population in each cell line and standard deviations among the replicates. **(e) **Expression of EMT-suppressing microRNAs (miRNAs) in the parental, A3, and F3 cells. qRT-PCR analyses were performed using specific primers to measure the expression of miR200c and miR205. At least three replications (*n *= 3) were performed to derive the average percentage of each cell population in each cell line and standard deviations among the replicates. Asterisks indicate statistical significance by two-tail *t *test (*P *< 0.05) for all quantitative results. ESA, epithelial-specific antigen.

Growing evidence demonstrates that many genes and proteins known to play essential roles during embryonic development are mutated or aberrantly expressed in cancerous cells. One of the most widely studied regulatory programs that controls normal developmental processes and contributes to tumor progression is the EMT [30,31]. Recently, it was shown that inducing EMT could generate mammary epithelial stem-like cells [15]. Furthermore, stem-like cancer cells exhibit EMT characteristics such as the loss of E-cadherin, increased expression of fibronectin and vimentin, and increased expression of mesenchymal transcription factors (Snail, Twist, and Slug) [32-34]. Therefore, we speculated that EMT plays a role in the increased tumorigenicity and metastatic potential of the cycling hypoxia-selected subpopulation. Indeed, we found specific molecular changes consistent with increased EMT in the cycling hypoxia-selected F3 subpopulations. MDA-MB 231 F3 and BCM2 F3 cells had an increased percentage of E-cadherin-negative cell population (8% to 10% increase) compared with the parental cell lines (Figure 6a; Figure S1 in Additional file 1). Within the E-cadherin-negative cell population, there was also an increased percentage of CD44^+^/CD24^- ^cell population in the F3 subpopulations (27% increase in MDA-MB 231 F3 and 35% increase in BCM2 F3) compared with the parental cell lines (Figure 6b). Since the loss of E-cadherin is a hallmark of increased EMT, the cycling hypoxia-selected F3 subpopulations appear to be enriched with stem-like and EMT cells.

Several transcription factors have been implicated in the transcriptional repression of E-cadherin. One of the first discovered and most important transcriptional repressors of E-cadherin is Snail [35]. Expression of Snail represses expression of E-cadherin and induces EMT in different cell types, including breast cancer cells [36,37]. Therefore, we speculated that molecular machinery found to promote EMT might also be upregulated in the cycling hypoxia-selected subpopulation. Consistent with a decrease in E-cadherin expression, MDA-MB 231 F3 and BCM2 F3 cells had increased nuclear expression of Snail and cytoplasmic expression of a mesenchymal marker vimentin (Figure 6c). Quantitative analysis of Snail and vimentin protein expression by Western blotting revealed a 3- to 4-fold increase in nuclear Snail expression and an 8- to 9-fold increase in vimentin expression in the MDA-MB 231 F3 and BCM2 F3 cells compared with the parental and hypoxia-exposed A3 subpopulations (Figure 6c). Furthermore, the cycling hypoxia-selected F3 subpopulations had increased mRNA levels of three mesenchymal transcription factors - Snail (7- to 10-fold), Slug (2- to 3-fold), and Twist (2- to 4-fold) - as measured by qRT-PCR (Figure 6d).

In addition to EMT-regulatory factors, regulatory molecules such as miRNAs can play a role in promoting stem-like and EMT phenotypes in cancer cells. Using a qRT-PCR-based method, we found dramatic decreases of epithelial miRNAs (miR200c and miR205) in the F3 subpopulations compared with the parental breast cancer cell lines (Figure 6e). It has been reported that human breast CSCs and normal human mammary stem/progenitor cells showed decreased expression of miR200c and other miR200 members and that restoring miR200c in breast CSCs inhibits their ability to expand clonally and form tumors *in vivo *[38]. Hence, downregulation of miR200c and miR205 in the cycling hypoxia-selected subpopulation corroborates with the stem-like phenotype exhibited in this subpopulation. In addition to miRNA200c and miR205, the cycling hypoxia-selected subpopulation showed decreased miR215 expression (Figure 6e). miR215 suppresses EMT by suppressing the mesenchymal transcription factor ZEB2 and increasing the E-cadherin level [39]. Therefore, downregulation of miR-215 could promote EMT phenotype in downregulation of E-cadherin and promote EMT phenotype in the cycling hypoxia-selected subpopulation. Together, our results reinforce the idea that EMT and stem cell factors work cooperatively in highly tumorigenic cancer subpopulations and raise interesting questions about their contribution to the increased metastatic capability observed in the cycling hypoxia-selected subpopulation.

## Discussion

Some studies have shown that external influences, such as hypoxia, can drive a 'reversible' phenotype that can enhance stem-like properties of cells to ensure survival of the tumor. Also, hypoxia-inducible factors have been shown to play a role in CSC self-renewal and tumor growth. It was reported, for example, that CSC state could be maintained by overexpressing hypoxia-inducible transcription factor (HIF2α) in glioma cell lines [40]. However, only few studies showed or discussed the possibility of establishing irreversible phenotypes through cycling hypoxic exposure without genetic manipulation [41,42]. In our study, we found that exposing metastatic breast cancer cell lines to hypoxia and reoxygenation cycles induces a unique subpopulation that is highly metastatic and exhibits stem-like and EMT phenotypes. Our results led us to speculate that there might be a metastatic subpopulation within stem-like cancer cells since other studies have shown that CSC phenotype alone may exhibit invasive property *in vitro *but is inadequate to determine or predict *in vivo *metastasis. Furthermore, we found that the stem-like and EMT phenotypes observed in the cycling hypoxia-selected subpopulation are not reversible, because we obtained the same molecular profile from this subpopulation by culturing them as spherical cultures for several months in normal oxygen content and culturing them as adherent cells (Figure S2 in Additional file 2).

Nevertheless, we believe hypoxia is only a partial driving force for the metastatic CSC enrichment. Our study showed that reoxygenation might also play a role in selecting stem-like breast cancer cells. The occurrence of reoxygenation following hypoxic exposures of various degrees is inherent in the dynamic nature of the tumor vasculature [43,44]. Fluctuating oxygen tensions in tumors could lead to reoxygenation-induced DNA damage and potentially increased genomic instability [45]. A hypoxia-dependent decrease in DNA repair could lead to the accumulation of unrepaired lesions in tumors and contribute to tumor progression [46]. A recent study by Pires and colleagues [23] showed that cells that are exposed acutely to hypoxia are able to restart replication regardless of the presence of active checkpoint response and reoxygenation-induced DNA damage. However, chronic exposure of cells to hypoxia induces disassembly of the replisome, preventing replication restart after reoxygenation [23]. In our study, we found that the emergence of a small non-adherent subpopulation (approximately 1%) survived after the first hypoxia/reoxygenation cycle and speculate that cyclic exposures of hypoxia and reoxygenation may select for the stem-like subpopulation with the ability to overcome replication arrest whereas the majority of non-adherent cells cannot. Also, as shown in our study, the surviving cycling hypoxia-selected subpopulation acquires additional molecular advantages after exposure to several cycles of hypoxia/reoxygenation.

Phillips and colleagues [47] reported that the non-adherent population of monolayer cultures of breast cancer cells has the ability to initiate mammosphere formation after irradiation. Other groups have also reported that exposing cancer cells to environmental factors such as serum deprivation or hypoxia alone can increase the stem-like phenotype or the number of stem-like cancer cells [40,48,49]. Collectively, our results support the idea that a stem-like subpopulation in the tumor could expand selectively in response to changes in the microenvironment. However, it is unclear whether the same stem-like subpopulation or a different one is generated by various conditions. To compare the non-adherent population described by Phillips and colleagues [47] and our F3 non-adherent subpopulation, we studied the expression level of Snail, an EMT transcription factor, in these two cell populations. Our results showed that the expression of Snail is robustly increased in cycling hypoxia-selected F3 cells compared with the non-nutrient-deprived floating cell population and the parental cell lines (Figure S2 in Additional file 2). Although our data indicate that these two cell populations are not the same, it is possible that this small viable floating cell population expands and gives rise to the F3 cell population after exposing cancer cells to cycling hypoxia and reoxygenation. We are currently exploring this possibility and hope to elucidate the mechanism and relevance of the cycling hypoxia-selected cell population in breast cancer progression.

## Conclusions

Although many studies have suggested the potential of CSCs as the seeds for distal metastasis, few studies have directly tested the metastatic capability of putative CSCs *in vivo*. Collective evidence from a few studies that directly tested the *in vivo *metastasis using sorted CSCs suggests that the CSC phenotype alone may exhibit invasive property *in vitro *but is not sufficient to determine or predict *in vivo *metastasis. Here, we show that a non-adherent, stem-like, and metastatic CSC-enriched subpopulation could be isolated by exposing human metastatic breast cancer cell lines to cycles of chronic hypoxia followed by reoxygenation. Since very few studies have demonstrated the formation of macro-metastasis from low numbers of sorted CSCs and currently proposed CSC markers might not be sufficient to identify all stem cell populations [13], we believe that our study presents a promising approach to isolate stem-like and metastatic breast CSCs as opposed to the cell-sorting strategy based on putative stem cell surface markers. Also, it will be of great interest to investigate the possibility that repetitive cycles of hypoxia/reoxygenation lead to the selective expansion of a pre-existing metastatic CSC subpopulation. Our results demonstrated the possibility of isolating highly metastatic breast CSCs using the hypoxia/reoxygenation regimen we established. With the recent success of identifying selective inhibitors targeting CSCs, we believe that the newly isolated cycling hypoxia-selected subpopulation may present a new opportunity for chemical screening and discovery of compounds with selective toxicity for metastatic breast CSCs.

## Abbreviations

CFE: colony-forming efficiency; CSC: cancer stem cell; EDTA: ethylenediaminetetraacetic acid; EMT: epithelial-mesenchymal transition; ESA: epithelial-specific antigen; MEM: minimum essential medium; MIRNA: microRNA; NOD/SCID: non-obese diabetic/severe combined immunodeficiency disease; PBS: phosphate-buffered saline; RQ: relative quantification; RT-PCR: reverse transcription-polymerase chain reaction; QRT-PCR: quantitative reverse transcription-polymerase chain reaction.

## Competing interests

The authors declare that they have no competing interests.

## Authors' contributions

EL helped to carry out the optimization of hypoxia/reoxygenation protocol, carried out the flow cytometry experiments and animal experiments, and prepared the manuscript. SC and MS helped to carry out the optimization of hypoxia/reoxygenation protocol. SN designed and performed the Western blot analysis and helped to design and perform tumor-sphere formation assays. CP helped to design and perform tumor-sphere formation assays. SP performed the real-time polymerase chain reaction analysis. XC performed the quantification of lung metastases. BS and JJ helped to perform and provide results for the miRNA analysis. EC helped to perform animal experiments and prepare the manuscript. All authors read and approved the final manuscript.

## Supplementary Material

Additional file 1 **Figure S1**. Gating parameters of ESA^+^/CD24^-^/CD44^+^, ESA^+^/CD24^+^/CD44^+^, E-cad^+^/CD24^-^/CD44^+^, and E-cad^+^/CD24^+^/CD44^+ ^cells using the Guava EasyCyte Flow Cytometer. Click here for file

Additional file 2 **Figure S2**. mRNA regulation of Snail in the parental, non-nutrient deprived (Flo), cycling hypoxia-selected cells grown in suspension culture (F3), and cycling hypoxia-selected cells grown in monolayer culture (F3 AD). Quantitative qRT-PCR analyses were performed using specific primers to measure the mRNA expression of the human Snail gene. Minimum three replications (*n *= 3) were performed to derive the average percentage of each cell population in each cell line and standard deviations among the replicates. Click here for file
