# Supplementary figures and images for: Identification of a stem-like cell population by exposing metastatic breast cancer cell lines to repetitive cycles of hypoxia and reoxygenation
Source: Breast Cancer Res. 2010 Nov 10;12(6):R94. doi: 10.1186/bcr2773 (PMC3046435; doi:10.1186/bcr2773)

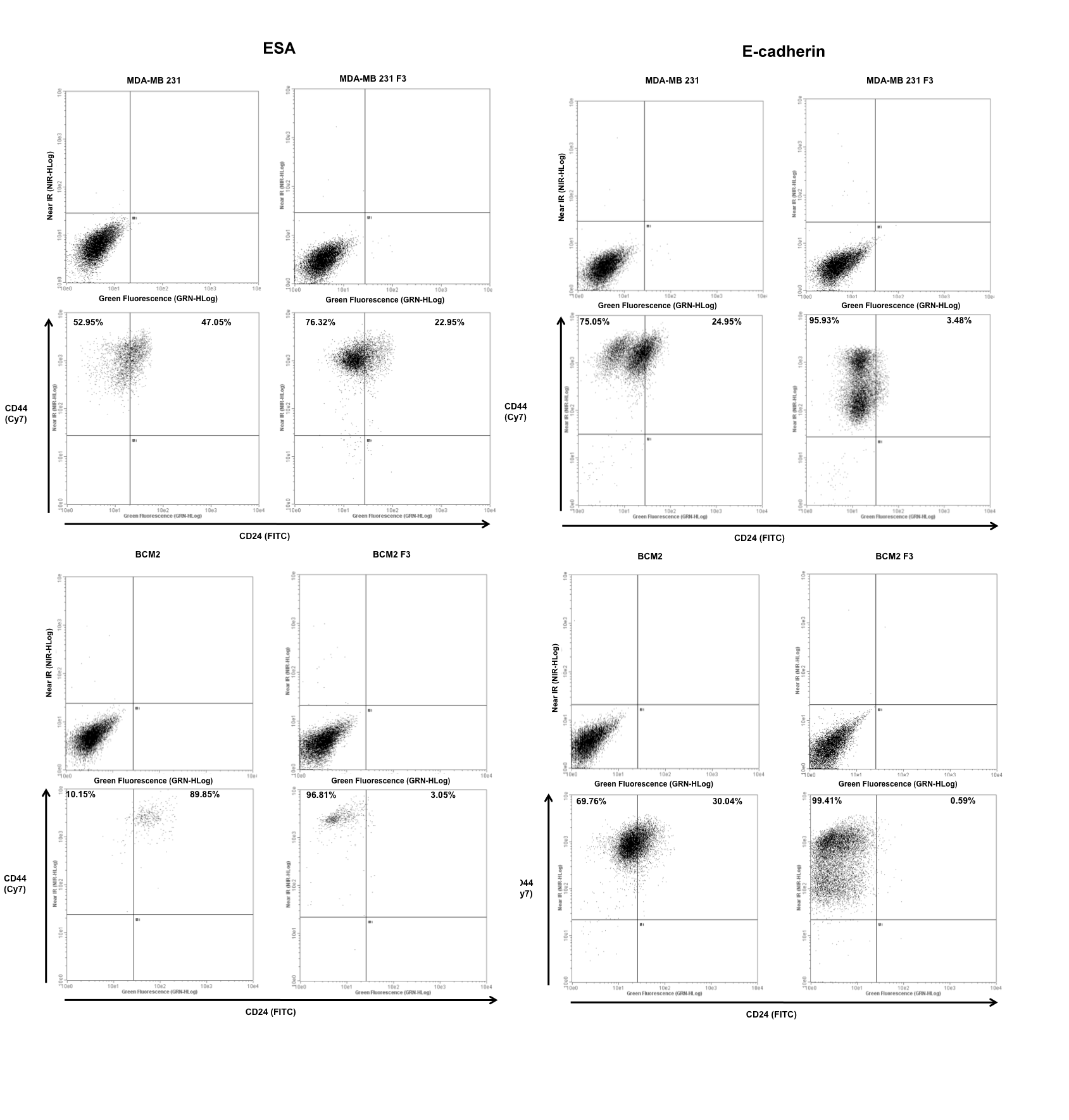

Supplement: Additional file 1 — Figure S1. Gating parameters of ESA+/CD24-/CD44+, ESA+/CD24+/CD44+, E-cad+/CD24-/CD44+, and E-cad+/CD24+/CD44+ cells using the Guava EasyCyte Flow Cytometer. [file bcr2773-S1.TIFF]

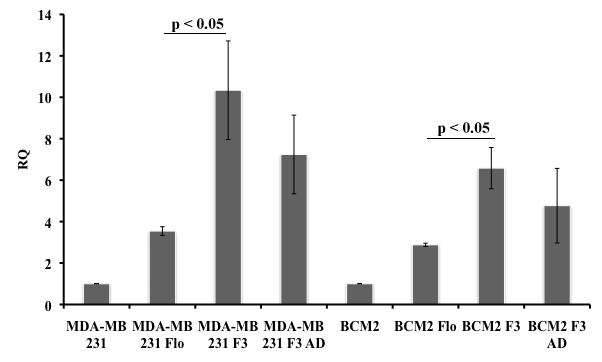

Supplement: Additional file 2 — Figure S2. mRNA regulation of Snail in the parental, non-nutrient deprived (Flo), cycling hypoxia-selected cells grown in suspension culture (F3), and cycling hypoxia-selected cells grown in monolayer culture (F3 AD). Quantitative qRT-PCR analyses were performed using specific primers to measure the mRNA expression of the human Snail gene. Minimum three replications (n = 3) were performed to derive the average percentage of each cell population in each cell line and standard deviations among the replicates. [file bcr2773-S2.TIFF]
